# Automatic Ankle Angle Detection by Integrated RGB and Depth Camera System

**DOI:** 10.3390/s21051909

**Published:** 2021-03-09

**Authors:** Guillermo Díaz-San Martín, Luis Reyes-González, Sergio Sainz-Ruiz, Luis Rodríguez-Cobo, José M. López-Higuera

**Affiliations:** 1 Photonics Engineering Group, University of Cantabria, 39005 Santander, Spain; luisrafael.reyes@unican.es (L.R.-G.); sergio.sainz@unican.es (S.S.-R.); miguel.lopezhiguera@unican.es (J.M.L.-H.); 2 CIBER-bbn, Instituto de Salud Carlos III, 28029 Madrid, Spain; luis.rodriguez@unican.es; 3 Instituto de Investigación Sanitaria Valdecilla (IDIVAL), 39011 Santander, Spain

**Keywords:** Kinect, IMU, ankle angle, depth camera, gait analysis, Mask RCNN, OpenPose

## Abstract

Depth cameras are developing widely. One of their main virtues is that, based on their data and by applying machine learning algorithms and techniques, it is possible to perform body tracking and make an accurate three-dimensional representation of body movement. Specifically, this paper will use the Kinect v2 device, which incorporates a random forest algorithm for 25 joints detection in the human body. However, although Kinect v2 is a powerful tool, there are circumstances in which the device’s design does not allow the extraction of such data or the accuracy of the data is low, as is usually the case with foot position. We propose a method of acquiring this data in circumstances where the Kinect v2 device does not recognize the body when only the lower limbs are visible, improving the ankle angle’s precision employing projection lines. Using a region-based convolutional neural network (Mask RCNN) for body recognition, raw data extraction for automatic ankle angle measurement has been achieved. All angles have been evaluated by inertial measurement units (IMUs) as gold standard. For the six tests carried out at different fixed distances between 0.5 and 4 m to the Kinect, we have obtained (mean ± SD) a Pearson’s coefficient, *r* = 0.89 ± 0.04, a Spearman’s coefficient, *ρ* = 0.83 ± 0.09, a root mean square error, RMSE = 10.7 ± 2.6 deg and a mean absolute error, MAE = 7.5 ± 1.8 deg. For the walking test, or variable distance test, we have obtained a Pearson’s coefficient, *r* = 0.74, a Spearman’s coefficient, *ρ* = 0.72, an RMSE = 6.4 deg and an MAE = 4.7 deg.

## 1. Introduction

The human gait analysis discipline has been highly developed in recent years with the base of new technologies. It can be applied in several areas, such as falls preventions [1,2], rehabilitation [3], sports [4], design of prosthetics [5], or design of robots [6]. 

Concretely, in medicine, which is the most significant area of investigation for gait analysis, it is essential to recognize different patterns in joint angles or distances to relating them to some anomaly or body deterioration and is closely associated with the prevention methods [7,8]. 

Therefore, several authors have searched for the gait analysis automatization using different technologies [9,10,11,12,13,14,15,16], such as Kinect v2 or accelerometers. In this sense, there are currently different degrees of development within the gait analysis in obtaining the different parameters with these new devices and methods. 

One of the critical parameters of gait analysis is ankle angle [17,18,19], due to its relation with spatial and temporal gait parameters as step and center of gravity during gait. According to related work, Kinect v2 has several problems detecting the ankle angle; in fact, the foot is the less accurate detected point, affecting the Kinect suitability as a tool for this analysis. However, according to [20], Kinect v2 can be used as a reliable and valid clinical measurement tool.

In this regard, one of the most advanced and widespread methods is the key point method, generally known as pose estimation, which consists of training algorithms to recognize specific points on the skeleton, such as the knee, elbow, neck, hip and others. Kinect v2 employs this method by default using a random forest-based algorithm that detects 25 points. More recently, new algorithms follow this idea, including OpenPose [21], which has developed another method using a convolutional neural network (CNN) to detect 25 points on the human body. On the other hand, another way consists of detecting the human body’s entire surface by generating a superimposed mask, and a region-based convolutional neural network (RCNN Mask) represents this method. From this overlay mask, the ankle angle is measured directly by projecting a line on the depth image and using linear regression to represent the foot and leg.

Based on the above, in this paper we will compare the results obtained by the Kinect’s algorithm, OpenPose algorithm, and the line projected on the mask, using depth and RGB images recorded with Kinect v2. As the gold standard, we use the angle measured by two IMUs through their Euler angles. 

## 2. Related Work

Due to the great importance of developing tools to prevent and detect the deterioration of the human body, both concerning older people and in rehabilitation within other areas, much work has been done in recent years. In this section, we review several publications related to the evaluation of the Kinect systems with gait analysis.

Kharazi (2015) [22] and Jamali and Behzadipour (2016) [23] employed a Kinect v1 for gait parameters and demonstrated good accuracy for some joints like knee and hip positions and angles, but they obtained poor results for ankle parameters.

In a comparison between Kinect v1 and v2 for joints accuracy, Wang (2015) [24] used an eight infrared stereo cameras system as a gold standard, shows that only the lower legs were tracked with large offsets in Kinect v2. Authors argue that it may be due to ToF technology’s use in its interaction with flat surfaces by generating noise. Another similar comparative was made by Moataz (2016) [25], who used an eight infrared camera motion analysis system and a single Kinect v2 sensor to measure gait parameters, and obtained similar results, with a high error for ankle angle measured by Kinect but good accuracy for others. 

Lamine (2017) [26] compares the gait analysis results from Kinect v2 with a Vicon motion system, with a person walking on a treadmill. The ankle angle obtained a mean error between 4° and 20°, with a Pearson correlation coefficient below 0.5.

Another interesting publication is Jeonghoon (2018) [27], in the first study to establish measurement characteristics during stair ambulation with Kinect v2. Authors show the Kinect v2 sensor’s ability to assess ankle joint kinematics was severely limited and comments that application of the technology for this purpose cannot be recommended at this time. 

More recently, Bilesan (2019) [28], in a comparison study between Kinect v2 and Vicon data for gait parameters, demonstrates one more time that ankle angle from Kinect v2 has low accuracy and it is not suitable as a measurement variable. 

Latorre (2019) [29], in a study in individuals with stroke, argues literally “parameters that involved ankle kinematics were discarded, as the Kinect v2 has been reported to have poor reliability in ankle detection”.

Some scientists have improved the Kinect’s internal algorithm based on random forest to solve some points’ low accuracy. These algorithms are called Human Pose Estimation. D’Eusanio (2016) [30] developed a neural network that improves the results obtained by the random forest in [31], but the foot joint only reaches 0.65% accuracy. Haque (2016) [32] employed a dataset containing 100 K annotated depth images to predict human joints in depth images. In the same way, Ballota (2018) [33] trained a fully convolutional network for human head detection in depth images. More recently, other authors have used OpenPose for gait analysis. Erika D’Antonio (2020) [34] used OpenPose to investigate the accuracy of such a system estimating kinematic parameters of human gait using two webcams synchronized with inconclusive results, commenting on the need for further analysis. Stenum (2020) [35] used a system based on RGB images recording a person from the side while walking to obtain gait parameters employing OpenPose, with high accuracy of 0.89 for ankle angle. 

Concerning the use of RCNN Mask with Kinect v2, Lee (2020) [36] uses this system to obtain human height estimation, reporting an error of 0.7%.

Due to all this information, authors have developed this method of projected lines to resolve the low accuracy in the ankle angle measurement from Kinect v2 and make it a more robust tool for gait analysis.

## 3. Materials and Methods

This work aims to obtain the person’s ankle angle employing the simultaneous recording with color and depth cameras, to improve the current methods to evaluate gait parameters. At this moment, with active cameras such as Kinect v2, it is possible to reach high precision measurements of other angles like the knee or the hips; however, the ankle angle is more complicated due to the low precision in the detection of the foot. In this paper, different detection methods are combined with depth data using projection lines. Therefore, it is a matter of obtaining, cleaning, processing, and verifying some of the Kinect information. The minimum experimental setup required to evaluate these methods is based only on a single active camera Kinect V2 and two inertial measurement units (IMUs) synchronized to a computer.

### 3.1. Tests 

All tests were carried out by one healthy person, aged 30, with no known abnormalities.

#### 3.1.1. Fixed Distance Tests

**Figure 1:**
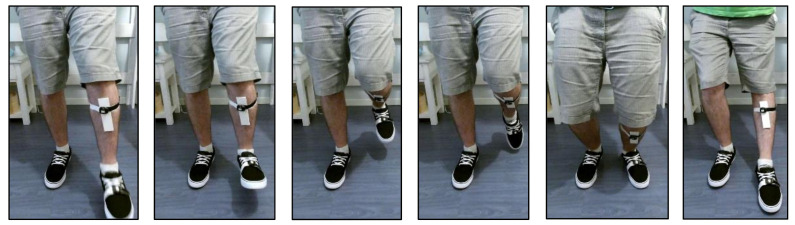
Example of frames from a recording of the Kinect v2 measuring simultaneously with the inertial measurement units (IMUs), one on the foot and one on the leg below the knee, for the calculation of the ankle angle. Distance of 0.5 m. Only shows the region of interest for each frame.

In these tests, one person is placed at a fixed distance from the Kinect and starts to make movements with the leg and foot to generate different ankle angles for about 30 s, as shown in the example in Figure 1. Distances are 0.5, 1, 1.5, 2, 3 and 4 m. 

#### 3.1.2. Walking Test

**Figure 2:**
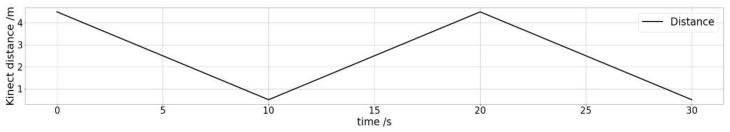
Distances covered during the walking test, always facing the camera.

In this test, one person walks from 4.5 m to less than one meter away, then moves back to his initial position, always facing the camera, and walks the same distance again. The test route is illustrated in Figure 2.

### 3.2. Experimental Setup

**Figure 3:**
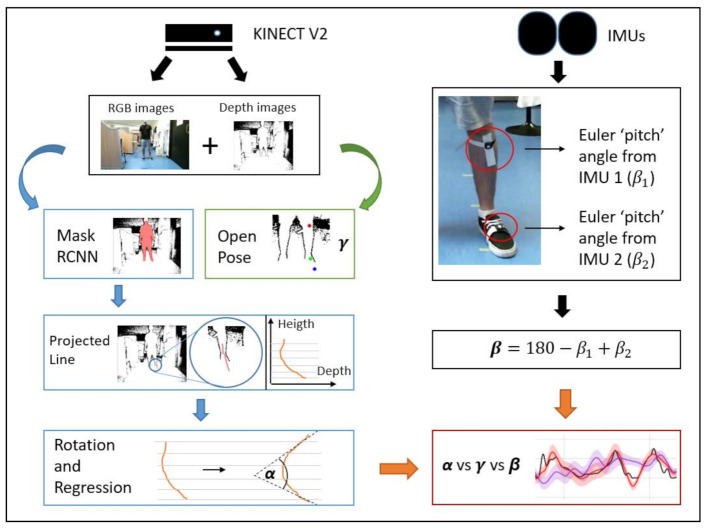
Scheme of the methods used to calculate the ankle angle using the Kinect v2 device with a region-based convolutional neural network (RCNN Mask) and OpenPose on the one hand, and two Inertial Measurement Units on the other.

The proposed methods to achieve automatic ankle angle detection rely on a relatively simple experimental setup, based on two primary sensors: an active camera Kinect v2 and two inertial measurement units (IMUs). A scheme of these proposed methods is depicted in Figure 3.

**Figure 4:**
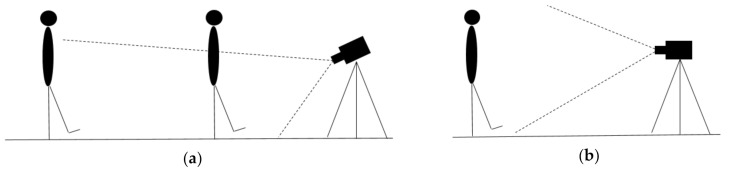
Different recording angles depending on the range of distances to be measured. (**a**) 0.5 and 1 m distance, as well as the walking test. (**b**) 1.5, 2, 3 and 4 m distances.

To carry out this experiment, the person’s foot must be within the Kinect field of view. Because of this, and in order to obtain the maximum possible distance range, the recording at 0.5 and 1 m, as well as the walking test, requires an inclination of the Kinect so that the upper body does not appear in the images. In contrast, this will not be necessary for the other distances, as illustrated in Figure 4. 

#### 3.2.1. Kinect v2-Integrated RGB and Depth Camera System

There are many devices that use depth cameras available on the market, and it is currently a growing technology. One of the employed devices in this experiment and one of the most employed devices of this kind from a general perspective is Microsoft Kinect v2. It works using time-of-flight (ToF) based technology (photons time-of-flight) to calculate each pixel’s depth, giving three coordinates *x*, *y*, *z* and time *t*. 

One of its main qualities is the integration of a body tracking system. Using a random forest algorithm, Kinect v2 can recognize the human body by giving each pixel different features with which it generates the classification of the 25 body joints [31]. It has a frequency of 30 Hz in both cameras and an RGB resolution of 1920 × 1080 px and a depth camera resolution of 512 × 424 px.

It is an affordable and easy-to-use device, being the main reason why it has been used in many studies that have to do with human body mobility, providing results that are considered adequate at a general level.

#### 3.2.2. Inertial Measurement Units for Angle Detection

Two inertial measurement units (IMUs) are employed to check the Kinect data quality; specifically, the MbiantLab Metamotion R IMU, selected for its performance and affordable price, which allows obtaining data in realtime and has a free App that will facilitate our work. One of the main tools is the direct receiving of Euler angles [37] from two simultaneously calibrated IMUs, which allows getting the angle between them quickly. These three Euler angles are those generated by rotating on each of the *x*, *y* and *z* axes, generally called “roll”, “pitch” and “yaw”, respectively. In our case, the sampling frequency of these angles is 100 Hz.

### 3.3. Methods

**Figure 5:**
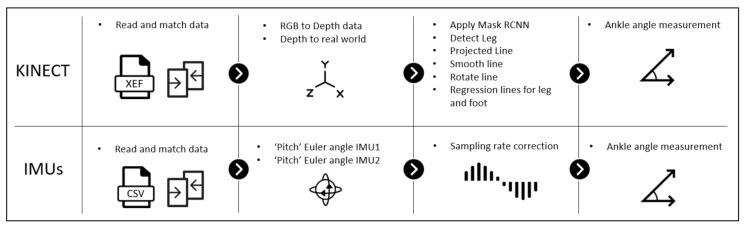
Steps to follow from reading the raw data to measuring the angle.

Based on the experimental setup described in the previous section, a specific processing scheme must be followed to maintain the whole process’s final accuracy. The relevant processing steps have been summarized in Figure 5 and are described in the following sections:

#### 3.3.1. Data Acquisition

This experiment is based on the recording, with an integrated RGB and depth camera system, of a person performing different movements with the lower limbs. He or she has two IMUs, one on the tibia and another on foot, to obtain the angles between them. The following explains the ways to get the data from Kinect and IMUs. 

To obtain a correct TimeStamp for the data, we connect all devices to the same computer simultaneously. 

Usually, Kinect v2 is placed at 1 m height to make the recording. The person is placed between 1.5 and 4.5 m, where Kinect can recognize the skeleton, and movements are performed. However, since we are experimenting with situations where the skeleton is not identified, the 1.5 m minimum distance limitation is not maintained. Instead, our range is extended to 0.5 m, in line with the limits of accuracy in the depth camera. 

Once the recording is done with KinectStudio 2.0 (specific software required to record the raw data coming from the active camera Kinect V2), a .xef file is generated, for which the KinectXEFTools tool has been used to read it. This tool generates a .avi file video with the RGB camera data and a .dat binary file with depth data from the IR camera. 

Depth data are 2d-arrays, one for each depth camera frame, with shape depth camera resolution (in Kinect v2 424 × 512 px) and its values are the distance of the pixel to the camera [38]. 

The RGB data is a video file from which we will extract each frame of 1920 × 1080 px for automatic recognition of the human body. 

It is also important to mention that although ideally both cameras have the same frequency, for processing requirements, this is not the case in practice. To minimize this problem, both sets of data have a timestamp for each frame to facilitate synchronization.

On the other hand, during the recordings the person wears the two IMUs on the leg and the foot. Self-adhesive strips have been used to place the IMUs on the body, tied around the leg and foot. A rigid platform has been placed on the leg strip, avoiding deviations mainly due to wrinkles in the clothes or something similar, but not on the foot because it makes it difficult to walk. 

We group all devices to be used in test in the same group, to collect Euler angles of all of them simultaneously, at 100 Hz each.

The first thing is to make sure that IMUs placed on the body have the same orientation; this means the IMU put on the leg at rest will have an Euler angle of approximately 90° to the ground, while the one on foot will be almost parallel to the floor and therefore an Euler angle of roughly 0°. The “pitch” angle is measured in these tests, with a range of −180° to 180°.

Data are sent to the computer in realtime and saved as CSV file through the MetaBase application.

**Figure 6:**
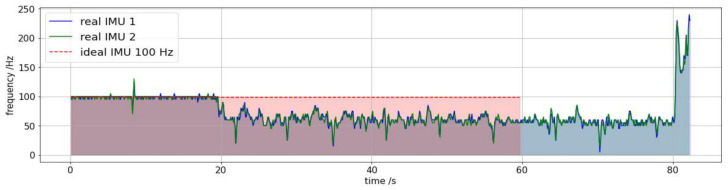
Real and ideal sampling rate of a IMUs experiment at 100 Hz with a computer that is simultaneously recording video with Kinect v2. The real signal must be corrected due to the lag that results from the lack of computer processing power.

However, it is noticed that this raw data could lead to a serious mistake. An issue was detected in the frequency at which the computer processed the data coming from the IMUs. This means that while the IMUs worked at about 100 Hz, and therefore sent 100 data per second via Bluetooth to the PC, the PC was not able to store that data so quickly since it was allocating most of its processing resources to the information it received from the Kinect via USB 2.0 and others process. The main evidence of this is depicted in Figure 6, in which the frequencies of the two IMUs are non-linear and have an exact match. 

**Figure 7:**
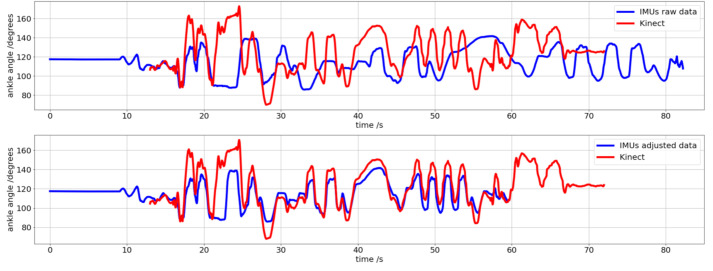
Real synchronization example for the IMUs and the Kinect before (**up**) and after (**bottom**) their adjustment.

To solve this anomaly, we proceed as follows: if it has taken 80 s to collect *N* samples at a non-constant frequency and if we accept that the error is coming from the computer, then we can easily calculate how long it has taken the IMUs to collect *N* samples at a 100 Hz constant frequency. Simply, $S_{r}=S_{i}$ where $S_{r}$ and $S_{i}$ are the area under the real and ideal curves in Figure 6, respectively. This step is essential to match the data from the IMUs to the Kinect, as can be seen in Figure 7.

#### 3.3.2. Body Recognition for RGB and Depth Images

One of the most important things to address in this experiment is body recognition in a recording video or pictures. To obtain a certain shape of the human body with an automatic method, we have employed a mask neural network and pose estimation software. 

**Figure 8:**
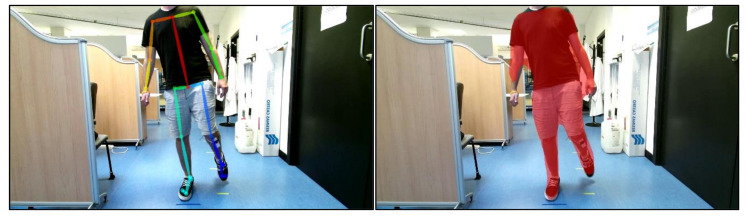
Frame of a recording to appreciate the correspondence between the human skeleton display by OpenPose (**left**) and the mask calculated by the Mask RCNN network (**right**).

Mask neural network passes through getting bounding boxes, which means a rectangular region where the detected object is founded to obtain its shape. Specifically, we have employed Mask RCNN with Keras module in Python, with pre-trained weights from the COCO dataset, where “person” is one of the classes trained. This neural network is based on [39], the Keras implementation can be consulted at [40], and an example is shown in Figure 8.

OpenPose is a pose estimation software based on Convolutional Neural Network, representing the human skeleton through 25 key points. This neural network is based on [21], the Keras implementation can be consulted at [41], and an example is shown in Figure 8.

Both methods run in RGB images. It is necessary to convert the pixels selected by the network to their corresponding pixels in the depth images to obtain the distance’s value in each one of them.

The first step is to synchronize each RGB image with its corresponding depth image. As mentioned above, the cameras’ frequencies are not the same and probably more images will be obtained from one camera than from another. Since the most important data is depth, the RGB image closest to it is selected for each depth array using each timestamp’s data. This way, we get the same number of depth images and color images.

Once achieved, RGB coordinates are converted to depth coordinates. For that, we know that the RGB camera has a resolution of 1920 × 1080 px, a focal length f = 3291 mm, and horizontal and vertical angles of view of 84.7° and 54.36°, respectively. 

**Figure 9:**
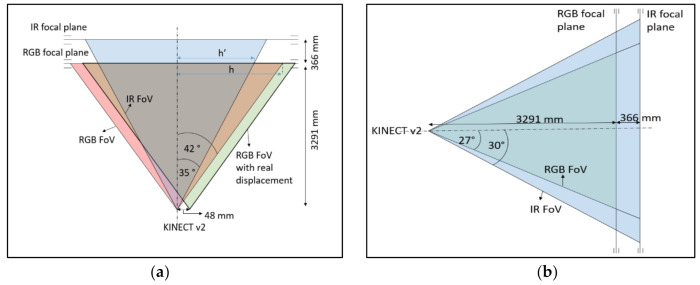
Diagram of the properties of the RGB and the depth cameras of the Kinect v2. (**a**) Properties of the horizontal field of view and focal plane distances for RGB and IR cameras. (**b**) Properties of the vertical field of view and focal plane distances for RGB and IR cameras.

Due to the two cameras’ different dimensions and focal lengths, not all the pixels in the RGB image exist too in their corresponding depth image. Assuming both cameras are located at the same point, the area that exists in both pictures is delimited, as shown in Figure 9.

To find the relation between both cameras coordinate systems, if we fix the origin at the center of the RGB focal plane with coordinates $\left(x,y\right)$, then$:\;x_{max}^{rgb}=960\;\mathrm{px}\;;\;x_{min}^{rgb}=-960\;\mathrm{px}$, and using the sine theorem and according to the scheme in Figure 8 obtain:

(1) $$h=3291\times \frac{\sin42}{\sin\left(90-42\right)}\;$$

(2) $$h^{\prime }=3291\times \frac{\sin35}{\sin\left(90-35\right)}\;$$

(3) h′h≈0.777 

So, it means that $x_{\max\_IR}^{rgb}=960\;\mathrm{px}*0.77=740\;\mathrm{px}$, being $x_{\max\_IR}^{rgb}$ the limit pixel x for which there is a corresponding pair in the depth image. In other words, all pixels x>740 and x<−740 in the RGB image do not exist in the depth image. In the images coordinate system, this means that $x^{IR}=512\;\mathrm{px}$ corresponds to $x^{RGB}=1700\;\mathrm{px}$ and $x^{IR}=0\;\mathrm{px}$ corresponds to $x^{RGB}=220\;\mathrm{px}$. To take into account the *x*-axis shift between the two cameras, simply divide h/960 ≅ 3$\;\frac{\mathrm{mm}}{\mathrm{px}}$. As the distance is 48 mm, the limits must be shifted to the left by 16 px. 

Analogously, for *y* pixel values, it is obtained that $x^{RGB}=0$ px corresponds to $x^{IR}=21\;\mathrm{px}$, and $x^{RGB}=1080\;\mathrm{px}$ corresponds to $x^{IR}=403\;\mathrm{px}$.

Therefore, if $P^{RGB}\;\left(x^{RGB},y^{RGB}\right)$ is a point in a color image, then the point $P^{IR}\;\left(x^{IR},y^{IR}\right)$ in the correspondent depth image is

(4) $$x^{IR}=\left(x^{RGB}-220-16\right)\times \frac{512}{1480}\;$$

(5) $$y^{IR}=21+y^{RGB}\times \frac{382}{1080}\;$$

**Figure 10:**
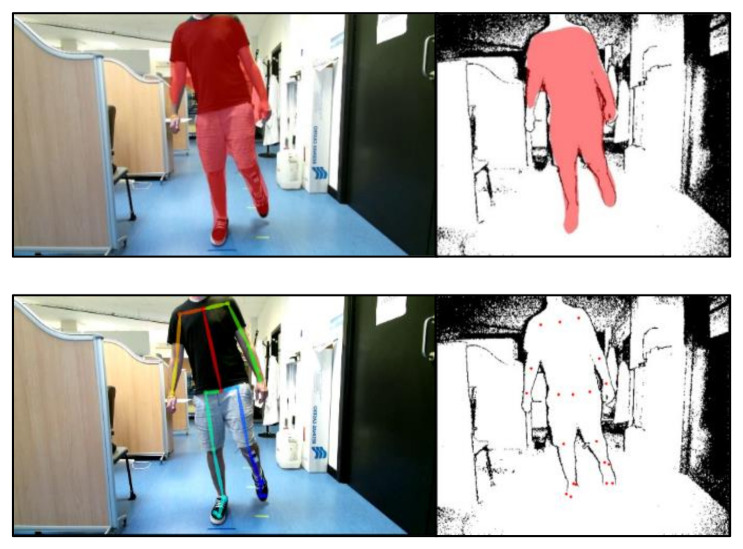
(**Up**) Correspondence between the mask in the RGB image and its calculated equivalent in the depth image. (**Down**) Correspondence between the OpenPose skeleton in the RGB image and its calculated equivalent in the depth image.

Now, we have a mask in the depth image that corresponds quite with the human shape, and we have the distance at which each pixel of that body is located. The same for the OpenPose data, as shown in Figure 10.

#### 3.3.3. Ankle Angle Measurement

To correctly measure the ankle angle, it is necessary to make another change of coordinates, specifically from the depth camera system to the real world system. For this task, we have all the data we need available.

The IR camera has a resolution of 424 × 512 px, a focal length *f* = 3657 mm, and horizontal and vertical angles of view of 70° and 60.2°, respectively.

These data indicate that the depth focal plane of the Kinect v2 is 5120 mm wide and 4240 mm high approximately, i.e., one pixel per centimeter. Using the sine theorem, we can quickly obtain the coordinates in the real world concerning the camera.

**Figure 11:**
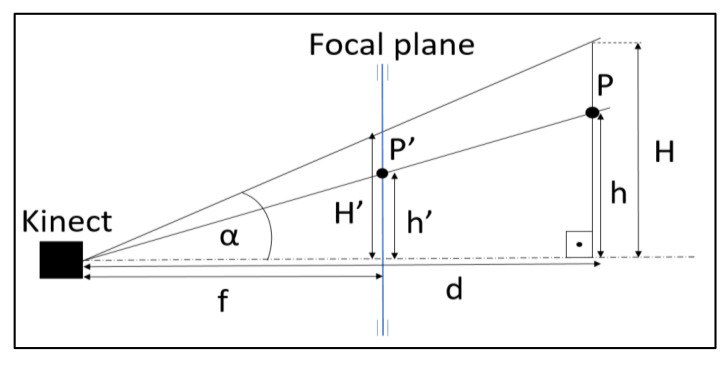
Relationship between the height of an object in the real world and the pixel of that object in a Kinect v2 depth image.

Following the scheme of Figure 11, with P’(x’, y’) being a point on a depth image and P being its real-world equivalent, the actual height of P, *h*, knowing that:

(6) $$\frac{h}{H}=\frac{h^{\prime }}{H^{\prime }}\;$$

(7) $$\frac{H}{\sin\alpha \;}=\frac{d}{\sin\left(90-\alpha \right)}\;$$

Then it is easy to obtain the h value, which follows the formula:

(8) $$h=h^{\prime }\times d\times \frac{\sin\alpha }{\sin\left(90-\alpha \right)}\times \frac{1}{H^{\prime }}\;$$

where $h^{\prime }=10\times y^{\prime }$, $H^{\prime }=$ 4240/2 mm, α=30.1° and d is the depth of the pixel P’, which is a known value. 

In this way, we obtain the point’s height, and the same logic is used to get the real lateral distance.

Therefore, if the point $P^{\prime }\;\left(x^{\prime },y^{\prime },\;z^{\prime }\right)$ is a depth pixel in an IR image, then the point $P\;\left(x,y,z\right)$ that corresponds to $P^{\prime }$ in the real world can be obtained as follows:

(9) $$z=z^{\prime }\;$$

(10) $$x=\;10\times x^{\prime }\times z\times \frac{\sin35}{\sin55}\times \frac{1}{2560}\;$$

(11) $$y=\;10\times y^{\prime }\times z\times \frac{\sin30.1}{\sin59.9}\times \frac{1}{2120}\;$$

Once the mask is generated in the depth image, the aim is to obtain the ankle position. 

Using OpenPose, and the same for the default Kinect skeleton, the problem is elementary, as we have located the three points (knee, ankle and foot) and their three *x*, *y*, *z* coordinates for each of them and each frame. With z being the depth and y the height, the angle γ is calculated using the known scalar product formula:

(12) $$\gamma =\arccos\left(\frac{z_{1}\times z_{2}+y_{1}\times y_{2}}{\sqrt{z_{1}^{2}+y_{1}^{2}}\times \sqrt{z_{2}^{2}+y_{2}^{2}}}\right)\;$$

with $z_{1}=z_{knee}-z_{ankle}$, $z_{2}=z_{foot}-z_{ankle}$,$\;y_{1}=y_{knee}-y_{ankle}$ and $y_{2}=y_{foot}-y_{ankle}$.

Regarding Mask RCNN, the process is more complex and is based on the projected line method, in which a line is projected on the leg of interest to know the ankle’s shape (Figure 9a). This projection line is generated from the mask, so it is essential to “clean” the mask to adjust it to the body as best as possible. We have implemented an algorithm to measure the average distance for all mask pixels in the depth image. With this distance, $\bar{d}$, we define an interval of minimum and maximum length where there can be parts of the body, in our case $d_{min}=\bar{d}-700$ and $d_{max}=\bar{d}+300$ in millimeters and then generate another more accurate “clean” mask. To select only the leg that we are interested in, calculate the minimum and maximum values for x in the mask, and select only the part we are interested in. Then calculate a height limit which must be below the knee, and that follows the formula lim= f×height, where f is an experimental value that depends on the distance,$\;\bar{d}$, according to Table 1.

**Figure 12:**
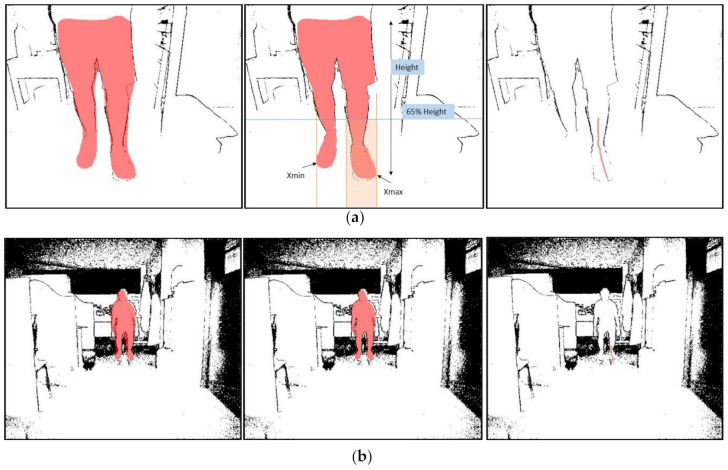
Example of two images processed to obtain the projection line on the ankle for (**a**) distance: 1 m (**b**) distance: 4 m. For each of one, it shows the mask transferred from the RGB image (**left**), the mask processed to improve the fit of the body by distance discrimination (**center**), and finally the resulting projection line (**right**).

Now, we can project a line across the leg, convert his coordinates to the real world and obtain the ankle angle. An example of these steps can be shown in Figure 12.

The project line contains points on the foot, the ankle and the low leg from the ankle until the knee. We obtain that the first point corresponds to the foot, the last point to the knee, and in the middle must be the ankle. For this experiment, the ankle is at the deepest point of the projection curve regarding the foot point and the knee point at the same time. The projection curve is rotated until the points foot and knee are at the same distance about the camera. The deepest point on the rotated curve is taken as a reference for the ankle.

Rotations follow the known matrix rotation for Euclidean space:

(13) $$\left[\begin{array}{c} x^{\prime }\\ y^{\prime } \end{array}\right]=\left[\begin{array}{cc} \cos\beta & -\sin\beta \\ \sin\beta & \cos\beta \end{array}\right]\left[\begin{array}{c} x\\ y \end{array}\right]\;$$

**Figure 13:**
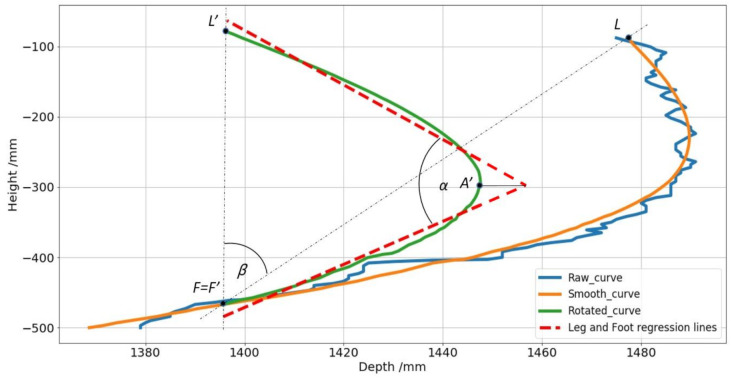
Graphic example showing the steps since the data obtained directly by the projection line (blue), the smoothed curve (orange) to the rotation line (green), that rotes beta degrees with the origin in the foot point, F, to keep the ankle as the furthest point to facilitate its recognition, A’. Finally, the regression lines (red) form the alpha angle, which is the angle of the ankle that is finally measured.

An example of this transformation is shown in Figure 13. In it, the irregular blue curve is the projection curve as we extract it. The orange curve that is superimposed on it is the same curve after smoothing with the Savitzky-Golay filter. The green curve is the same as the orange one rotated one β angle so that L (leg) and F (foot) are at the same depth. This is done so that regardless of the camera’s position about the leg, the ankle is always the deepest point, making it easier to locate it approximately. The measurement of the ankle angle, α, is done by simple linear regression of each part $F^{\prime }-A^{\prime }$ and $A^{\prime }-L^{\prime }$. 

For the IMUs, once data collection is done, we have Elapsed time, TimeStamp and Euler angles for each of one. Same as with the Kinect cameras, the first step is to synchronize both devices. Even if IMUs data were collected simultaneously at the same frequency, there will always be a time lag between one IMU data and the other one in the real world. This lag is due to multiple factors intrinsic to their materiality and the element’s processing capabilities. Thus, it is essential to perform a re-synchronization task using data from two different IMUs to obtain the right angle.

**Figure 14:**
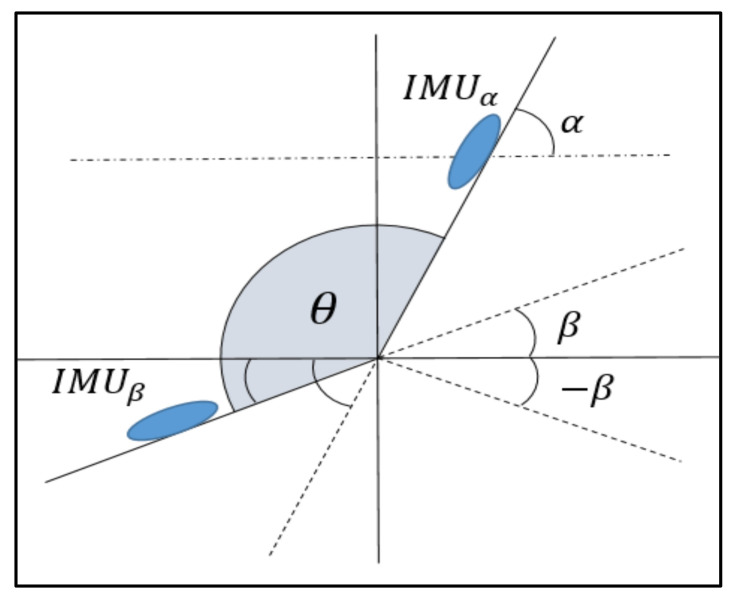
Graphic example to measure the angle formed by two IMUs, *θ*, through their Euler *α* and *β* ‘pitch’ angles.

Following the scheme in Figure 14, if the $IMU_{\alpha }$ is on the leg below the knee, and the $IMU_{\beta }$ is on foot, then the ankle angle, θ, is calculated as:

(14) $$\theta_{ankle}=180-\alpha_{leg}+\beta_{foot}\;$$

This formula is valid for all other possible situations with $\alpha ,\;\beta \;\in \;\left[-180,180\right]$.

#### 3.3.4. Statistics

Pearson’s correlation coefficient (*r*) was used to assess the association’s linear strength between the two motion capture methods. For the remaining non-normally distributed parameters, Spearman’s rho was used instead. For both coefficients, their 95% confident intervals were calculated using Fisher’s Z score, $r_{z}$:

(15) $$r_{z}\;\pm \;z_{\frac{\alpha }{2}}\times se\;$$

where $se=\frac{1}{\sqrt{N-3}}$ and $z_{\frac{\alpha }{2}}$ is the Z value for the 95% CI in the Standart Normal Distribution Table, and it has been calculated with *scipy* package using $z_{\frac{\alpha }{2}}=\;stats.norm.ppf\left(1-\frac{\alpha }{2}\right)$.

To provide sufficient information on one device’s measurements to the other, the root means squared error (RMSE) and the mean absolute error (MAE) have been calculated.

(16) $$RMSE=\sqrt{\frac{\sum_{n=1}^{N}{\left(x_{K,n}-x_{I,n}\right)}^{2}}{N}}\;$$

(17) $$MAE=\frac{\sum_{n=1}^{N}\left|x_{K,n}-x_{I,n}\right|}{N}\;$$

where $x_{K}$ is each Kinect angle value, $x_{I}$ is its corresponding IMUs angle value, and N is the total number of measurements.

#### 3.3.5. Propagation of Uncertainty

Analytically, operating only with the depth distance error, disregarding the rest, since the angle is obtained employing the scalar product, and assuming that the depth at the same frame is the same for the knee and foot:

(18) $$\cos\alpha \;=\frac{z_{k}\times z_{f}+y_{k}\times y_{f}}{\sqrt{z_{k}{}^{2}+y_{k}{}^{2}}+\sqrt{z_{f}{}^{2}+y_{f}{}^{2}}}=\frac{z^{2}+y_{k}\times y_{f}}{\sqrt{z^{2}+y_{k}{}^{2}}+\sqrt{z^{2}+y_{f}{}^{2}}}=g\left(z\right)\;$$

where $z_{k}$ is the knee depth and $z_{f}$ is the foot depth, assuming that $z_{k}=z_{f}=z$, with the origin in the ankle depth. Operating only with the depth distance error and disregarding the rest, then:

(19) $$\alpha =arccos\left(g\left(z\right)\right)=f\left(z\right)\;$$

(20) $$\delta \alpha =\frac{df\left(z\right)}{dz}\times \delta z\;$$

with [38]:

(21) $$\delta z=\frac{0.01}{3}z+\frac{0.025}{3}\;$$

The IMUs have an error of less than a tenth of a degree, so they are considered measures without error.

## 4. Results

This section shows the results obtained for measuring the ankle angle according to the criteria and techniques explained in the previous sections.

### 4.1. Distance Tests

First, the results of six recordings at different distances from the Kinect camera are presented below to cover the entire range of distances allowed by the device. These distances are 0.5, 1, 1.5, 2, 3 and 4 m. As for the 0.5 m distance test, Kinect algorithm and OpenPose library cannot recognize the human body, so there are no skeleton metrics. All results have been calculated using the methods detailed in the statistics section.

Numerical results of the metrics used are shown in Table 2 and Table 3. In this table, we offer the data referring to our method of projection lines against the standard of IMUs, and also the values obtained by the skeleton that generates the Kinect automatically when it exists. Pearson and Spearman’s coefficients have been calculated with their corresponding 95% confidence intervals, and all *p*-values are lower than 0.0001.

**Figure 15:**
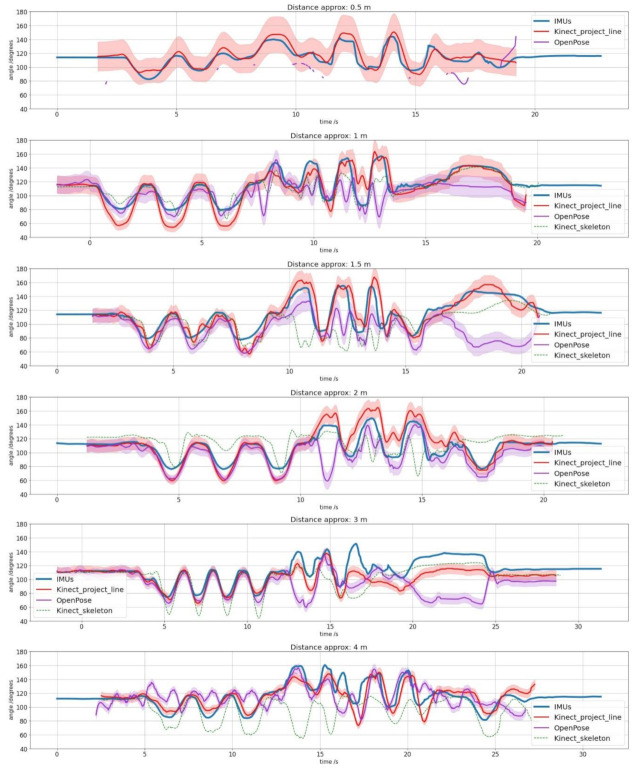
Graph showing the comparison between the ankle angle measured by the Kinect v2 with our projection lines method (red), the ankle angle measured by OpenPose (violet) and the angle measured by the IMUs using Euler’s angles (blue), for different distances in relation to the Kinect, which are: 0.5, 1, 1.5, 2, 3 and 4 m. Red and violet shaded area is the Kinect angle error following Equation (20). In addition, when it exists, the ankle angle measured with the default skeleton generated by the Kinect (green) is also represented.

Figure 15 shows the value of the ankle angles measured by the Kinect by projection line, by pose estimation using OpenPose library and by the IMUs as a function of time. When it exists, the ankle angle formed by the skeleton automatically generated by the Kinect is also shown to compare results.

**Figure 16:**
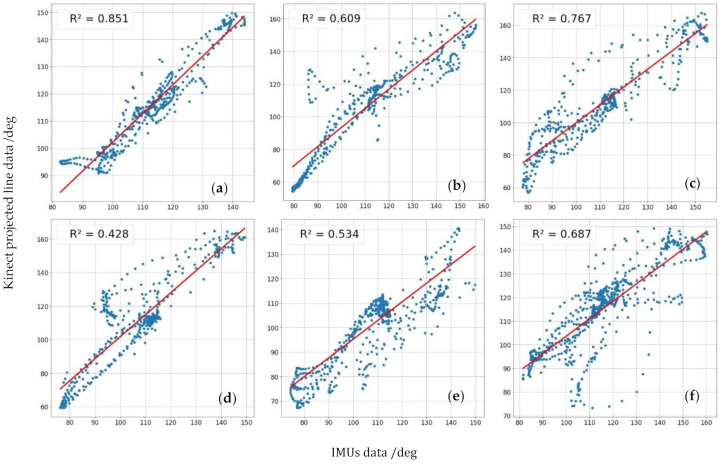
Graph showing the linearity between the ankle angle measured by the Kinect v2 with our method of projection lines and the angle measured by the IMUs using Euler’s angles, for different distances in relation to the Kinect, which are: (**a**) 0.5, (**b**) 1, (**c**) 1.5, (**d**) 2, (**e**) 3 and (**f**) 4 m.

Finally, with the same intention of comparing our method’s values with the IMUs Euler angle’s as gold standard, Figure 16 shows the values of one method against the other to see how the relationship between them works and better understand the results of Table 2.

### 4.2. Walking Test

**Figure 17:**
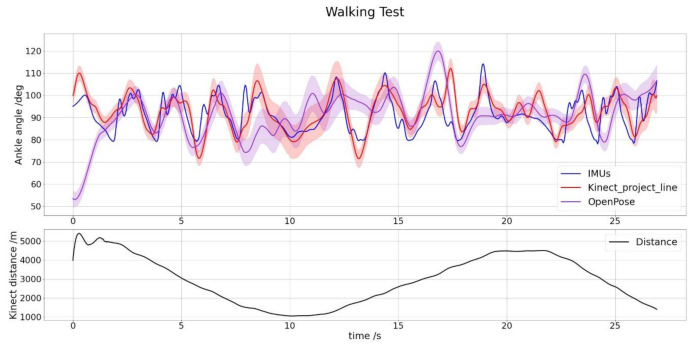
Graph showing the comparison between the ankle angle measured by the Kinect v2 with our projection lines method (red), the ankle angle measured by OpenPose (violet) and the angle measured by the IMUs using Euler’s angles (blue), for the walking test. Red and violet shaded area is the Kinect angle error following Equation (20). The body distance average is shown below to complete the information.

**Figure 18:**
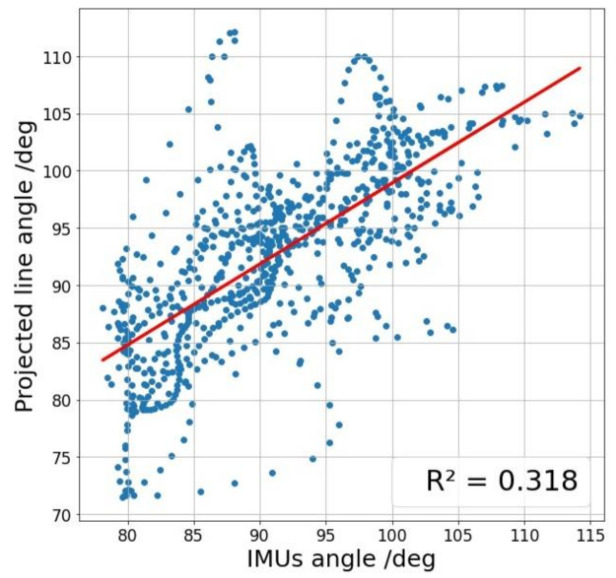
Graph showing the linearity between the ankle angle measured by the Kinect v2 with our method of projection lines and the angle measured by the IMUs using Euler’s angles, for different a walking test between 5 and 1 m.

Second, the walking test results are shown below. Kinect algorithm cannot recognize the human body, so there are no default skeleton metrics (see Figure 4). Measured metrics are the same as distance tests and can be seen in Table 4 and Table 5 and Figure 17 and Figure 18.

## 5. Discussion

Table 2 and Table 4 show a robust linear relationship, which means that the relative variations of the angles are very similar, so the measured values’ differences are almost constant and easily adjustable. The six fixed distance tests obtain a Pearson’s coefficient average between our method and the standard measurement with IMUs of 0.89, which means a solid and stable linear relationship between the two, and obtains its maximum of 0.95 at the minimum distance where neither Kinect nor OpenPose is not able to recognize the person. Spearman’s coefficients give us useful information when evaluating the results since they allow us to observe subsets of non-linear measurements within the whole set. Therefore, we can detect frames in which the projected line method measurement and the IMUs are not in agreement. For example, this occurs in the fifth image of Figure 16, a distance equal to 3 m. It is observed that the intermediate values follow a slight concave curve within the overall linearity of the data set. The six tests’ average obtains a Spearman’s coefficient of 0.83, slightly lower than Pearson’s but which in absolute terms also reflects a strong relationship of monotony between the two curves and obtains its maximum of 0.94 at the same distance which Pearson’s coefficient is greater 0.5 m. For the walking test, correlation coefficients suffer a significant decline, with 0.74 and 0.72 values for Pearson and Spearman, respectively. One of the causes could be wearing long jeans because they cover the ankle and modify the projection line’s profile. Other possible technical reasons will have to be analyzed when using this method in future investigations of all kinds. However, the correlations obtained offer promising results. We believe that the moving average used and the linear regression representing foot and leg smooth out the trousers’ peaks that the Kinect can measure in some pixels, improving the system’s accuracy.

Regarding error metrics, it has been considered to include both the RMSE and the MAE because of the criteria diversity and to provide more information. For the fixed distance tests, the mean of the RMSE is 10.3°, and the mean of the absolute error is 7.5, in a range of 360° for the six tests. These values demonstrate high precision in mean value calculation for an ankle angle recording. Together with the correlation coefficients, as can be seen in Figure 14, our results provide a high precision not only for the mean value but for each value calculated in each frame. Similar results are obtained in the walking test, in which the mean of the RMSE is 6.39°, and the mean of the absolute error is 4.6. 

We want to stress how important it is that the Kinect has obtained the best values at that distance where it is complicated to generate automatic recognition with pose estimation technics. At this distance of 0.5 m, the RMSE is 5.5°, the MAE is 4.4°, the r-squared show in Figure 15 is 0.851 and Pearson’s and Spearman coefficients are 0.95 and 0.94, respectively. With this data, our method demonstrates its ability to make accurate measurements at distances of less than one meter, even though the Kinect error at that distance is more significant. Therefore, this method is suitable for use in places with little space for testing with angle measurements.

In contrast, the OpenPose library has obtained results below the method proposed in this paper, and in many cases, worse than Kinect’s random forest-based algorithm. It is an unexpected result, with 0.54 and 0.5 values for Pearson and Spearman, respectively, in the fixed distance tests, and 0.25 and 0.26, respectively, for the walking test. One of the possibilities is that the successive unit conversions, mainly between RGB and depth, can significantly affect a very narrow area such as the ankle. A few pixels difference can move the “ankle” point located in the RGB image outside the human body in the depth image. The Kinect algorithm uses depth data to identify the pixel corresponding to the ankle and therefore does not have this problem. Another possibility is that, although the ankle’s RGB pixel is correctly translated to the depth pixel, its position is not exact. OpenPose detects the ankle with a margin of error and sometimes places it on foot, sometimes on the leg, and this alters the angle that is finally measured with Equation (12). OpenPose indeed recognizes the ankle angles generated by bending and rising well, as are the first few seconds of each fixed distance test, which are easily recognized by their bell-shaped design. However, other types of movements that are not accompanied by the rest of the body are more difficult to measure.

Regarding the comparison with the Kinect’s default skeleton, the results indicate that our method practically doubles the angle measurement precision. Furthermore, it is much more robust with similar results in all tests, while the default skeleton varies significantly in its metrics. For example, if it has achieved good results for the distance of one meter, even improving our method’s values in the RMSE and the MAE, the results are awful for the 1.5 and 4 m, and for the rest, they are still worse than ours. Therefore, our method has proven to be much more accurate and robust than the default skeleton.

In turn, there are two significant limitations of our method. The first is that this method is not a realtime method. The time spent processing Kinect data of a recording video for about one minute is approximately half an hour, so it could be a problem in situations in which results must be obtained faster. Authors think this problem can be resolved if instead use the Kinect Studio .xef file for processing data, and we work with a code that reads each frame information in realtime and calculates the angle for each one. However, this leads to a slight decrease in the data acquisition frequency, and it is a future research way for the authors. 

The second limitation is relative to *f* (height) factor in Table 1. This experimental factor can need few variations according to the person’s size and the Kinect angle in relation to the person, which are the variables that define the proportion of the body that appears in the image. Nevertheless, the *f* factor adaptation is straightforward and can be calibrated using a single frame for each distance to be measured. Similarly, the angle measurement differences between IMUs and the projected line method are because the person is bent over, and the projection line has exceeded the knee’s height. This makes the project line take up points at a greater distance, and this problem can be observed in the valleys of Figure 15, mainly for distances of 1 and 2 m. The solution for this limitation is simple, and it consists of placing the Kinect at a lower height perpendicular to the area to be measured.

Concerning the scope of the tests, the seven measurements were carried out in our laboratory and on a single healthy person, which act as “reference” for the method validation under normal conditions. Thus, the results cannot be fully generalized at this point; further testing of the method on a larger number of subjects is necessary. We hope to continue exploring the limits of the proposed method in future publications.

On the one hand, all these arguments and data lead us to corroborate the projection line method’s validity and superiority accuracy, using an integrated RGB and Depth camera system, over current pose estimation, due to its capacity to generate a real relief for each frame and its ability to measure at distances of less than one meter. On the other hand, pose estimation methods are faster.

## 6. Conclusions

The present paper results for ankle angle extraction from Kinect v2 using a RCNN Mask network demonstrate that the projection lines method is highly accurate. Comparing this method and the golden standard using Inertial Measurement Units shows significant correlation and precision, highly improving the results reported by Kinect’s own algorithm and OpenPose library, both of them pose estimation technics.

This method can be applied to improve gait analysis when using devices similar to Kinect v2, providing new and accurate data. Authors are also confident this same method can be used any device that integrates a color camera and a depth camera, only by adapting the camera resolutions and lens properties to that particular device.

The adaptability of this method is extensive and its application depends strongly on the object detection capability of the recently developed algorithms. For areas where the algorithms have low precision, this technique can be of great help and can be used in many fields of study as the RCNN Mask network currently detects 80 different classes. With this technique, angles and distances of such objects could easily be measured even in small environments, as demonstrated in this manuscript. 

The next aim is to be able to perform the projection on other objects to detect more variables and reduce the calculation times as much as possible.

## Figures and Tables

**Table 1:** Relationship between distance and height factor, *f*.

Distance/m	*f*
0.5	0.65
1	0.65
1.5	0.75
2	0.8
3	0.9
4	0.95

**Table 2:** In distance tests, correlation coefficients obtained by the projection lines method based on RCNN Mask proposed in this work, OpenPose software and the Kinect default skeleton, versus the IMUs gold standard. All Pearson’s and Spearman’s *p*-values < 0.0001.

	Pearson, r [95%CI]			Spearman, ρ [95%CI]		
**Distance/m**	**Projected Line**	**OpenPose**	**Default Skeleton**	**Projected Line**	**OpenPose**	**Default Skeleton**
0.5	0.95 (0.94, 0.96)	-	-	0.94 (0.93, 0.95)	-	-
*1*	0.90 (0.88, 0.91)	0.60 (0.55, 0.66)	0.86 (0.84, 0.88)	0.90 (0.88, 0.91)	0.60 (0.54, 0.65)	0.89 (0.87, 0.90)
*1.5*	0.92 (0.90, 0.93)	0.61 (0.55, 0.67)	0.19 (0.10, 0.28)	0.91 (0.89, 0.92)	0.66 (0.60, 0.71)	0.33 (0.25, 0.41)
*2*	0.89 (0.87, 0.91)	0.74 (0.70, 0.77)	0.33 (0.25, 0.41)	0.75 (0.71, 0.78)	0.75 (0.71, 0.78)	0.50 (0.43, 0.56)
*3*	0.85 (0.83, 0.87)	0.28 (0.22, 0.35)	0.74 (0.70, 0.77)	0.67 (0.63, 0.71)	0.18 (0.11, 0.25)	0.57 (0.51, 0.61)
*4*	0.83 (0.80, 0.85)	0.43 (0.37, 0.49)	0.28 (0.21, 0.35)	0.85 (0.83, 0.87)	0.25 (0.18, 0.32)	0.38 (0.31, 0.44)
Mean ± SD	0.89 ± 0.04	0.53 ± 0.15	0.4 ± 0.2	0.83 ± 0.09	0.5 ± 0.2	0.5 ± 0.2

**Table 3:** In distance tests, error values obtained by the projection lines method based on Mask RCNN proposed in this work, OpenPose software and the Kinect default skeleton, versus the IMUs gold standard.

	RMSE/deg			MAE/deg		
Distance/m	Projected Line	OpenPose	Default Skeleton	Projected Line	OpenPose	Default Skeleton
***0.5***	**5.50**	-	-	**4.40**	-	-
***1***	13.40	19.10	**12.20**	9.90	11.71	**8.22**
***1.5***	**10.50**	24.20	28.80	**7.70**	16.90	17.40
***2***	**13.60**	16.50	19.10	**10.00**	10.72	11.30
***3***	**10.69**	23.40	14.90	**7.49**	14.53	9.70
***4***	**10.60**	18.37	30.70	**7.40**	14.50	21.00
***Mean ± SD***	***10 ± 2***	*20 ± 3*	*20 ± 4*	***7.5 ± 1.8***	*13 ± 2*	*13 ± 7.*

**Table 4:** In walking test, correlation coefficients obtained by the projection lines method based on RCNN Mask proposed in this work, OpenPose software and the Kinect default skeleton, versus the IMUs gold standard. All Pearson’s and Spearman’s *p*-values < 0.0001.

Pearson, r (95% CI)			Spearman, ρ [95%CI]		
**Projected Line**	**OpenPose**	**Default Skeleton**	**Projected Line**	**OpenPose**	**Default Skeleton**
0.74 (0.78,0.70)	0.25 (0.20, 0.30)	-	0.72 (0.68, 0.74)	0.26 (0.20, 0.32)	-

**Table 5:** In walking test, error values obtained by the projection lines method based on Mask RCNN proposed in this work, OpenPose software and the Kinect default skeleton, versus the IMUs gold standard.

RMSE/deg			MAE/deg		
Projected Line	OpenPose	Default Skeleton	Projected Line	OpenPose	Default Skeleton
6.39	11.75	-	4.76	8.63	-

## Data Availability

Data available on request due to privacy restrictions.

## References

[1] Tinetti M.E., Speechley M., Ginter S.F. (1988). Risk Factors for Falls among Elderly Persons Living in the Community. N. Engl. J. Med..

[2] Tinetti M.E., De Leon C.F.M., Doucette J.T., Baker D.I. (1994). Fear of Falling and Fall-Related Efficacy in Relationship to Functioning Among Community-Living Elders. J. Gerontol..

[3] Baker R. (2006). Gait analysis methods in rehabilitation. J. Neuroeng. Rehabil..

[4] Gouwanda D., Senanayake S.M.N.A. Emerging Trends of Body-Mounted Sensors in Sports and Human Gait Analysis. Proceedings of the 4th Kuala Lumpur International Conference on Biomedical Engineering.

[5] Gard S.A. (2006). Use of Quantitative Gait Analysis for the Evaluation of Prosthetic Walking Performance. JPO J. Prosthetics Orthot..

[6] Hao S., Dawei W., Rongjie K., Yan C. (2018). Gait analysis and control of a deployable robot. Mech. Mach. Theory.

[7] Bridenbaugh S.A., Kressig R.W. (2010). Laboratory Review: The Role of Gait Analysis in Seniors’ Mobility and Fall Prevention. Gerontology.

[8] Woollacott M., Shumway-Cook A. (2002). Attention and the control of posture and gait: A review of an emerging area of research. Gait Posture.

[9] Dubois A., Bihl T., Bresciani J.-P. (2017). Automating the Timed Up and Go Test Using a Depth Camera. Sensors.

[10] Steinert A., Sattler I., Otte K., Röhling H., Mansow-Model S., Müller-Werdan U. (2019). Using New Camera-Based Technologies for Gait Analysis in Older Adults in Comparison to the Established GAITRite System. Sensors.

[11] Capecci M., Ceravolo M.G., Ferracuti F., Iarlori S., Longhi S., Romeo L., Russi S.N., Verdini F. Accuracy evaluation of the Kinect v2 sensor during dynamic movements in a rehabilitation scenario. Proceedings of the 2016 38th Annual International Conference of the IEEE Engineering in Medicine and Biology Society (EMBC).

[12] Paolini G., Peruzzi A., Mirelman A., Cereatti A., Gaukrodger S., Hausdorff J.M., Della Croce U. (2013). Validation of a Method for Real Time Foot Position and Orientation Tracking With Microsoft Kinect Technology for Use in Virtual Reality and Treadmill Based Gait Training Programs. IEEE Trans. Neural Syst. Rehabil. Eng..

[13] Tan D., Pua Y.-H., Balakrishnan S., Scully A., Bower K.J., Prakash K.M., Tan E.-K., Chew J.-S., Poh E., Tan S.-B. (2018). Automated analysis of gait and modified timed up and go using the Microsoft Kinect in people with Parkinson’s disease: Associations with physical outcome measures. Med. Biol. Eng. Comput..

[14] Liu L., Mehrotra S. Patient walk detection in hospital room using Microsoft Kinect V2. Proceedings of the 2016 38th Annual International Conference of the IEEE Engineering in Medicine and Biology Society (EMBC).

[15] Cippitelli E., Gasparrini S., Spinsante S., Gambi E. (2015). Kinect as a Tool for Gait Analysis: Validation of a Real-Time Joint Extraction AlgorithmWorking in Side View. Sensors.

[16] Geerse D., Coolen B., Kolijn D., Roerdink M. (2017). Validation of Foot Placement Locations from Ankle Data of a Kinect v2 Sensor. Sensors.

[17] Lin P.-Y., Yang Y.-R., Cheng S.-J., Wang R.-Y. (2006). The Relation Between Ankle Impairments and Gait Velocity and Symmetry in People With Stroke. Arch. Phys. Med. Rehabil..

[18] Buck P., Morrey B.F., Chao E.Y. (1987). The optimum position of arthrodesis of the ankle. A gait study of the knee and ankle. J. Bone Jt. Surg. Am..

[19] Seel T., Raisch J., Schauer T. (2014). IMU-Based Joint Angle Measurement for Gait Analysis. Sensors.

[20] Otte K., Kayser B., Mansow-Model S., Verrel J., Paul F., Brandt A.U., Schmitz-Hübsch T. (2016). Accuracy and Reliability of the Kinect Version 2 for Clinical Measurement of Motor Function. PLoS ONE.

[21] Cao Z., Hidalgo Martinez G., Simon T., Wei S.-E., Sheikh Y.A. (2019). OpenPose: Realtime Multi-Person 2D Pose Estimation using Part Affinity Fields. IEEE Trans. Pattern Anal. Mach. Intell..

[22] Kharazi M.R., Memari A.H., Shahrokhi A., Nabavi H., Khorami S., Rasooli A.H., Barnamei H.R., Jamshidian A.R., Mirbagheri M.M. Validity of Microsoft KinectTM for measuring gait parameters. Proceedings of the 22nd Iranian Conference on Biomedical Engineering(ICBME 2015), Iranian Research Organization for Science and Technology (IROST).

[23] Jamali Z., Behzadipour S. (2016). Quantitative evaluation of parameters affecting the accuracy of Microsoft Kinect in GAIT analysis. Proceedings of the 2016 23rd Iranian Conference on Biomedical Engineering and 2016 1st International Iranian Conference on Biomedical Engineering (ICBME).

[24] Wang Q., Kurillo G., Ofli F., Bajcsy R. Evaluation of Pose Tracking Accuracy in the First and Second Generations of Microsoft Kinect. Proceedings of the 2015 International Conference on Healthcare Informatics.

[25] Eltoukhy M., Oh J., Kuenze C., Signorile J. (2017). Improved kinect-based spatiotemporal and kinematic treadmill gait assessment. Gait Posture.

[26] Lamine H., Bennour S., Laribi M., Romdhane L., Zaghloul S. (2017). Evaluation of Calibrated Kinect Gait Kinematics Using a Vicon Motion Capture System. Comput. Methods Biomech. Biomed. Eng..

[27] Oh J., Kuenze C., Jacopetti M., Signorile J.F., Eltoukhy M. (2018). Validity of the Microsoft Kinect™ in assessing spatiotemporal and lower extremity kinematics during stair ascent and descent in healthy young individuals. Med. Eng. Phys..

[28] Bilesan A., Owlia M., Behzadipour S., Ogawa S., Tsujita T., Komizunai S., Konno A. (2018). Marker-based motion tracking using Microsoft Kinect. IFAC-PapersOnLine.

[29] Latorre J., Colomer C., Alcañiz M., Llorens R. (2019). Gait analysis with the Kinect v2: Normative study with healthy individuals and comprehensive study of its sensitivity, validity, and reliability in individuals with stroke. J. Neuroeng. Rehabil..

[30] D’ Eusanio A., Pini S., Borghi G., Vezzani R., Cucchiara R. (2019). Manual Annotations on Depth Maps for Human Pose Estimation. Mathematics and Computation in Music.

[31] Shotton J., Sharp T., Kipman A., FitzGibbon A., Finocchio M., Blake A., Cook M., Moore R. (2013). Real-time human pose recognition in parts from single depth images. Commun. ACM.

[32] Haque A., Peng B., Luo Z., Alahi A., Yeung S., Fei-Fei L. (2016). Towards Viewpoint Invariant 3D Human Pose Estimation. Machine Learning and Knowledge Discovery in Databases, Proceedings of the Applied Data Science and Demo Track, Amsterdam, The Netherlands, 11–14 October 2016.

[33] Ballotta D., Borghi G., Vezzani R., Cucchiara R. Fully Convolutional Network for Head Detection with Depth Images. Proceedings of the 2018 24th International Conference on Pattern Recognition (ICPR).

[34] D’Antonio E., Taborri J., Palermo E., Rossi S., Patane F. A markerless system for gait analysis based on OpenPose library. Proceedings of the 2020 IEEE International Instrumentation and Measurement Technology Conference (I2MTC).

[35] Stenum J., Rossi C., Roemmich R. (2020). Two-dimensional video-based analysis of human gait using pose estimation. Biorxiv.

[36] Lee D.-S., Kim J.-S., Jeong S.C., Kwon S.-K. (2020). Human Height Estimation by Color Deep Learning and Depth 3D Conversion. Appl. Sci..

[37] Junkins J.L., Shuster M.D. (1993). The Geometry of the Euler Angles. J. Astronaut. Sci..

[38] Pagliari D., Pinto L. (2015). Calibration of Kinect for Xbox One and Comparison between the Two Generations of Microsoft Sensors. Sensors.

[39] He K., Gkioxari G., Dollár P., Girshick R. (2018). Mask R-CNN. arXiv.

[40] (2018). Mask R-CNN for Object Detection and Instance Segmentation on Keras and TensorFlow. https://github.com/matterport/Mask_RCNN..

[41] Hidalgo G., Cao Z., Simon T., Wei S.-E., Raaj Y., Joo H., Sheikh Y. https://github.com/CMU-Perceptual-Computing-Lab/openpose.

